# New aspects of longitudinal instabilities in electron storage rings

**DOI:** 10.1038/s41598-018-30306-y

**Published:** 2018-08-09

**Authors:** A. Blednykh, B. Bacha, G. Bassi, W. Cheng, O. Chubar, A. Derbenev, R. Lindberg, M. Rakitin, V. Smaluk, M. Zhernenkov, Yu-chen Karen Chen-Wiegart, L. Wiegart

**Affiliations:** 1 Brookhaven National Laboratory, Upton, NY 11973 USA; 2 Advanced Photon Source, Argonne National Laboratory, Argonne, IL 60439 USA; 3 Department of Materials Science and Chemical Engineering, Stony Brook University, Stony Brook, NY 11794 USA

## Abstract

Novel features of the longitudinal instability of a single electron bunch circulating in a low-emittance electron storage ring are discussed. Measurements and numerical simulations, performed both in time and frequency domain, show a non-monotonic increase of the electron beam energy spread as a function of single bunch current, characterized by the presence of local minima and maxima, where a local minimum of the energy spread is interpreted as a higher-order microwave instability threshold. It is also shown that thresholds related to the same zero-intensity bunch length depend linearly on the accelerating radio frequency voltage. The observed intensity-dependent features of the energy spread, confirmed by measurements with two independent diagnostics methods, i.e. horizontal beam profile measurements by a synchrotron light monitor and photon energy spectrum measurements of undulator radiation, are given a theoretical interpretation by applying a novel eigenvalue analysis based on the linearized Vlasov equation.

## Introduction

Successful construction, commissioning and operation of several ultra-low emittance storage rings have had a decisive impact on the development direction of future synchrotron light source facilities. For example, PETRA-III, NSLS-II, and, recently, MAX-IV, have all demonstrated reliable operation with a horizontal electron beam emittance equal to or smaller than 1 nm-rad^1–3^. While these and the new generation of storage ring light sources such as the ESRF-EBS^4^, APS-U^5^, ALS-U^6^ and Sirius^7^ significantly reduce the horizontal emittance, they do not offer any improvement of another important part of the full 6-dimensional electron beam emittance, namely, the energy spread. The electron beam energy spread can limit the peak harmonic flux from undulators, particularly at the high harmonics that are extensively used in medium-energy storage rings. In addition, the energy spread affects the angular divergence of the emitted radiation, and may ultimately limit the x-ray brightness in future ultra-low-emittance storage ring based light sources. Therefore, it is important to have a detailed understanding of what determines the electron beam energy spread in storage ring facilities under a variety of operating conditions.

The energy spread of a low-current electron beam in a storage ring is determined by the equilibrium between radiation damping and quantum fluctuations^8^. As the beam current increases, the intra-bunch particle interaction via short-range wakefields induced by the beam in a vacuum chamber modifies the longitudinal particle distribution as a function of the beam intensity (see, e.g.^9,10^). The energy spread is not changed by the collective effects if the beam intensity is low enough, but the longitudinal microwave instability occurs above a certain threshold current and results in the energy spread growth and in the beam brightness degradation. This instability typically manifests itself as a high-frequency perturbation of the beam that increases both the energy spread and bunch length until a new quasi-equilibrium state is established. One of the goals of a storage ring design is to keep the instability threshold above the operating current.

In this paper, we study the onset of the microwave instability and its behavior well above the threshold current using precise measurements at the NSLS-II storage ring^11^. Our measurements show that the energy spread growth is not monotonic with the beam current, but rather is characterized by a number of local minima and maxima. In addition, spectral analysis of the beam motion show that as the energy spread goes through a local minimum, the oscillation frequency of the perturbation changes almost discontinuously by a value comparable to the synchrotron frequency. Thus, we find evidence for the higher-order instability thresholds, and observe this general phenomenology over a wide range of accelerating radio frequency (RF) voltages and electron bunch lengths. We then reproduce the energy spread growth and spectral features with the simulation code  SPACE^12^, and finally compare the measurements and simulations to a recently developed theory extending Sacherer’s^13,14^ insights to interpret these microwave instability thresholds in terms of a succession of classical mode coupling. We find that the predictions based on the mode coupling theory are quite successful and appear to provide a useful way for better understanding of the microwave instability over a wide range of beam parameters.

The main results of this paper hinge upon experimental data for the energy spread *σ*_*δ*_ as a function of single-bunch current *I*_0_ in the NSLS-II storage ring, where *δ* = (*E* − *E*_0_)/*E*_0_ is the relative energy deviation with respect to the reference energy *E*_0_, and *I*_0_ = *Q*/*T*_0_, where *Q* is the bunch charge and *T*_0_ is the revolution period. Two independent diagnostic methods have been applied to measure the current-dependent energy spread *σ*_*δ*_(*I*_0_)^15,16^. The first method is based on measurements of horizontal beam profile using a synchrotron light monitor (SLM) installed in a low-dispersion area^17^, where the energy spread *σ*_*δ*_ is related to the horizontal beam size *σ*_*x*_ as^18^:

1 $${\sigma }_{\delta }({I}_{0})=\sqrt{{\sigma }_{x}^{2}({I}_{0})-{\beta }_{x}{\varepsilon }_{x}}/{\eta }_{x},$$

where *ε*_*x*_ is the horizontal emittance; *β*_*x*_ and *η*_*x*_ are, respectively, the horizontal beta function and dispersion in the bending magnet, in which the beam generates light detected by the SLM. In our case, *β*_*x*_ = 2.77 m and *η*_*x*_ = 0.13 m. The second method is based on the measurement of photon energy spectrum from the in-vacuum undulator (IVU) of the Soft Matter Interfaces (SMI) X-ray beamline^19^.

In addition to the energy spread measurements, spectra of the beam motion were also measured using a stripline, which is a part of NSLS-II bunch-by-bunch transverse feedback system^20,21^. This study of longitudinal beam dynamics in the spectral domain allows us to characterize the current-dependent behavior of the synchrotron frequency and its harmonics above the first threshold of microwave instability.

NSLS-II is a *E*_0_ = 3 GeV storage ring with the revolution period *T*_0_ = 2.6 *μ*s and the momentum compaction *α*_*c*_ = 3.7 × 10^−4^. The storage ring is equipped with three damping wigglers (DWs) designed to control the horizontal beam emittance and energy spread. If the magnet gaps of the DWs are open, the horizontal emittance and energy spread are determined by the “bare” magnet lattice (BL): *ε*_*x*_ = 2 nm, *σ*_*δ*_ = 0.05%. For user operations, the gaps of all three DWs are closed to achieve the design emittance *ε*_*x*_ = 0.9 nm^22^ and the energy spread *σ*_*δ*_ = 0.087%. We denote this magnet lattice as 3DW. For the 3DW lattice with RF voltage *V*_RF_ = 3 MV, the bunch length is *σ*_*z*0_ = 4.6 mm and the synchrotron tune is *ν*_*s*0_ = 8.67 × 10^−3^ in the limit of *I*_0_ → 0. The reduction of the horizontal beam emittance by DWs and corresponding changes in the energy spread and beam lifetime have been confirmed experimentally^2^.

**Figure 1:**
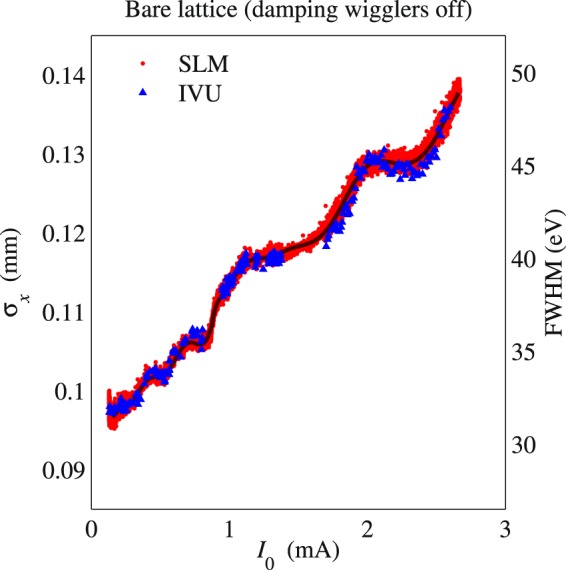
Horizontal beam size measured by the SLM and the measured FWHM of the SMI IVU spectrum at the 7^th^ harmonic. These measurements have been done with *V*_RF_ = 2.6 MV for the bare lattice.

**Figure 2:**
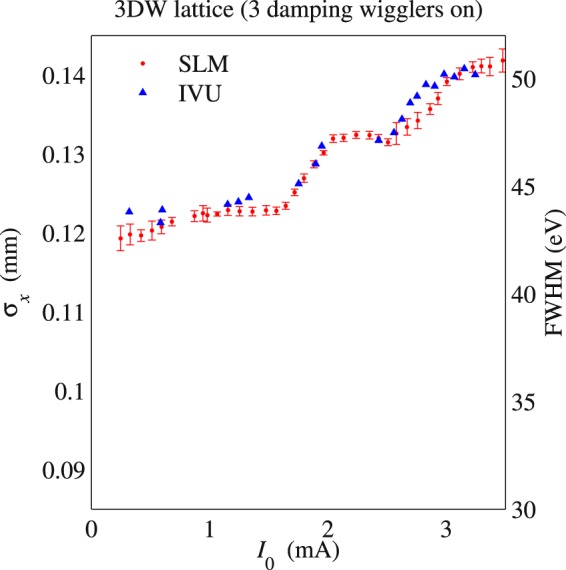
Horizontal beam size measured by the SLM and the measured FWHM of the SMI IVU spectrum at the 7^th^ harmonic. These measurements have been done with *V*_RF_ = 2.6 MV for the 3DW lattice.

The *σ*_*δ*_ dependence on the number of DWs is illustrated by the different low-current values of the measured *σ*_*x*_ in Fig. 1 for the bare lattice and in Fig. 2 for the 3DW lattice. The RF voltage was *V*_RF_ = 2.6 MV in both cases. Using Eq. (1) to extract the energy spread from *σ*_*x*_, one finds that the measurements agree with the design values of 0.087% for the 3DW lattice and 0.05% for the BL, in the limit *I*_0_ → 0. More important, Figs. 1 and 2 show good agreement between independent measurements of two parameters dependent on the energy spread: horizontal beam size and harmonic width of undulator radiation. In particular, *σ*_*x*_ measured by the SLM is superimposed on the full width at half-maximum (FWHM) of the 7^th^ harmonic of the SMI IVU radiation spectra. Both sets of data closely agree over the whole range of *I*_0_, and the measured growth of both *σ*_*x*_ and the IVU FWHM are characterized by the presence of local minima and maxima at certain values of the beam current. This non-monotonic behavior is of particular interest, and we will use the measurements, simulations, and theory to show that they arise when different modes of oscillation become unstable as the beam current increases.

At the nominal NSLS-II operation mode (3DW), the spectral measurements of the 7^th^ harmonic of radiation from 23 mm period and 2.8 m long IVU of the SMI beamline demonstrated about 16% larger harmonic width than this was predicted by simulations. This systematic effect is attributed to a possible small misalignment of this undulator with respect to electron beam^23^ and a detector effect. Measurements with another undulator, 20 mm period and 3 m long IVU of Coherent Hard X-ray (CHX) scattering beamline, performed in parallel with the measurements at the SMI, were in better agreement with simulations (within 1%), however, these measurements were done using a slower detection system, that required exposure times exceeding the electron beam lifetime at high currents per bunch. Therefore, only the SMI data were included in this paper. We note that the single-electron spectral width of the 7^th^ harmonic of the SMI IVU at 8.1 keV photon energy is about 9.5 eV, which contributes only a few percent to the measured harmonic width (assuming validity of the quadratic summation rule for different factors contributing to the width).

**Figure 3:**
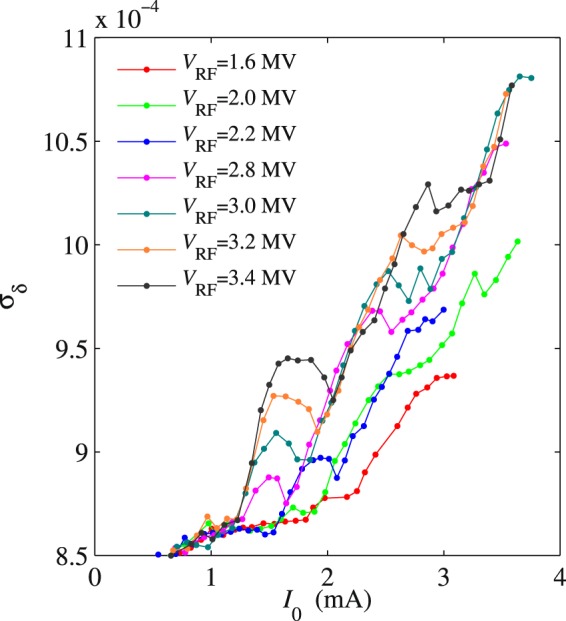
Indirect measurements of *σ*_*δ*_ as a function of *I*_0_ at different *V*_RF_ obtained from the SLM data for the 3DW lattice. The error-bars are similar to those shown in Fig. 2, but have been omitted here for clarity.

In Fig. 3, we plot the *σ*_*x*_ dependence on *I*_0_ for the 3DW lattice and various RF voltages. The energy spread is derived from the SLM measurements of *σ*_*x*_(*I*_0_) using Eq. 1. As mentioned previously, both measurements in Fig. 3 and the theory predict *σ*_*δ*_(0) = 0.087% in the low-current limit. This value was also confirmed experimentally by measurements of decoherence of betatron oscillation^24^. Below a (RF voltage-dependent) threshold current, which determines the first threshold of microwave instability, the energy spread is approximately constant and equal to its unperturbed value, as shown in Fig. 3. The slight *σ*_*δ*_ drift below *I*_0_ = 0.5 mA is a systematic error caused by the diagnostic setup of SLM for *σ*_*x*_ measurements. Above the threshold current, the microwave instability induces an energy spread increase that may be significant even at low beam intensity. The energy spread increase above the first microwave instability threshold is modulated by a number of local maxima and minima of *σ*_*δ*_. At any RF voltage, the difference between the peaks and valleys becomes smaller as *I*_0_ increases, and we will refer to the currents at which *σ*_*δ*_ reaches a local minimum as higher-order thresholds (in our terminology, the “first threshold” constitutes the onset of the microwave instability). It is interesting to note that for a given RF voltage these higher-order thresholds approximately make a straight line, and that the slope $d{\sigma }_{\delta }/d{I}_{0}$ of this line increases with *V*_RF_.

**Figure 4:**
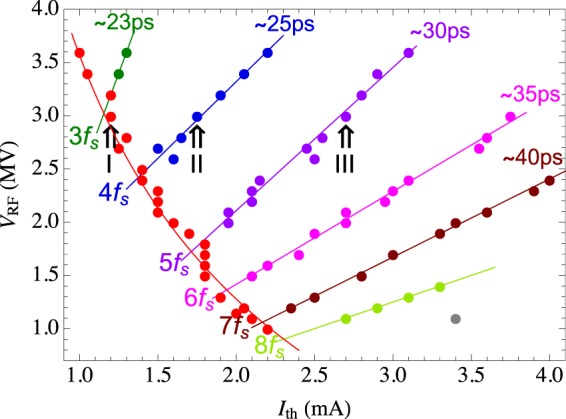
Locations of the measured instability threshold currents for different *V*_RF_. Red dots represent the first threshold *I*_*th*1_, the red curve is a fit. The other color-coded dots show the measured local minima of *σ*_*δ*_: the dots with the same color correspond approximately to the same measured bunch length *σ*_*z*_ and perturbation frequency, and the integer part of that frequency is as indicated.

Figure 4 summarizes the instability thresholds depending on the RF voltage. Each dot in Fig. 4 indicates where the minima of *σ*_*δ*_ were observed in the $({I}_{0},{V}_{{\rm{RF}}})$ plane. The red dots represent the measured values of the first microwave instability threshold, while the red solid curve is a fit. Note that the red points show small oscillations around the fitting curve, with the largest excursions occurring when the first and second thresholds approach each other at ${V}_{{\rm{RF}}}=3.2$ MV, ${V}_{{\rm{RF}}}=2.6$ MV, ${V}_{{\rm{RF}}}=1.8$ MV and ${V}_{{\rm{RF}}}=1.4$ MV. For the beam currents above the first microwave instability threshold, the local minima of *σ*_*δ*_ are naturally grouped according to the measured bunch length, so that the points with the same color in Fig. 4 correspond approximately to the same bunch length. As an example, we highlighted the first, second, and third order instability thresholds at ${V}_{{\rm{RF}}}=3$ MV, where the thresholds labeled “I”, “II”, and “III” occur at ${I}_{th}=1.2$ mA, ${I}_{th,{\rm{II}}}=1.8$ mA, and ${I}_{th,{\rm{III}}}=2.7$ mA, respectively. The measurement results shown in Fig. 3 are highly repeatable, and have been reproduced during several study periods separated by few weeks.

To interpret theoretically the measurements of the intensity dependence of the energy spread we distinguish three different bunch intensity regimes: 1) the low-intensity, or single-particle dynamics regime, where intensity-dependent effects are negligible; 2) the medium-intensity, or bunch-lengthening regime, where the dynamics is characterized by a stationary distribution with a constant energy spread and intensity-dependent bunch length; 3) the high-intensity, or microwave instability regime, where both energy spread and bunch length exhibit a time and intensity-dependent behavior.

In the first, low-intensity regime, the measured horizontal beam size depends upon the lattice functions *β*_*x*_ and *η*_*x*_ and on the equilibrium values of the energy spread and emittance, the latter two of which are determined by the single-particle process of incoherent emission of synchrotron radiation. The time evolution of the electron phase space density in the low-intensity regime is governed by the Fokker-Planck equation^25^, which consists of a Hamiltonian part determined by the lattice configuration, and by a non-Hamiltonian part that describes the damping and diffusion due to the emission of synchrotron radiation. In the second, medium-intensity regime, the dynamics is changed by the addition of a longitudinal wakefield that is produced by the electromagnetic interaction of the electron bunch with its surroundings. The medium-intensity dynamics is similar to that of the low-intensity regime, with the same equilibrium energy spread but with a longitudinal profile that is modified by the wakefield.

The microwave instability threshold current delimits the transition from the second regime to the third, high-intensity regime. Above the threshold current the energy spread increases with current, and the collective dynamics is governed by the time-dependent Vlasov-Fokker-Planck equation. Our goal is to characterize the transition to, and dynamical behavior within, the third regime.

The Vlasov-Fokker-Planck equation for the evolution of the phase space density Ψ is^26^

2 $$\frac{\partial {\rm{\Psi }}}{\partial \tau }+p\frac{\partial {\rm{\Psi }}}{\partial q}-[q+{Y}_{c}F(q,\,{\rm{\Psi }},\,\tau )]\frac{\partial {\rm{\Psi }}}{\partial p}=\alpha \frac{\partial }{\partial p}(p{\rm{\Psi }}+\frac{\partial {\rm{\Psi }}}{\partial p}),$$

where the longitudinal coordinates *q* and *p* are normalized with respect to the bunch length *σ*_*z*_ and energy spread *σ*_*δ*_ respectively, i.e. $q=z/{\sigma }_{z}$ and $p=\delta /{\sigma }_{\delta }$, where *z* is the longitudinal distance with respect to the reference particle. In Eq. (2), $\tau ={\omega }_{s}t$ is used in place of time *t* as independent variable, where ${\omega }_{s}=2\pi {f}_{s}$ is the synchrotron frequency, and $\alpha =\mathrm{2/(}{\omega }_{s}{\tau }_{s})$, where *τ*_*s*_ is the longitudinal radiation damping time.

Here ${Y}_{c}=e{I}_{0}/({E}_{0}{\omega }_{s}{\sigma }_{\delta })$, $F(q,{\rm{\Psi }},\tau )={\int }_{-\infty }^{q}dq^{\prime} {W}_{\parallel }(q-q^{\prime} )\lambda (q^{\prime} ,\tau )$, where *W*_||_(*q*) is the longitudinal wakefield and $\lambda (q,\tau )=\int dp{\rm{\Psi }}(q,p,\tau )$ is the longitudinal density. Equation (2) is solved numerically with the particle tracking code SPACE^12^ using a longitudinal wakefield *W*_||_ that was obtained numerically using the code GdfidL^27^. For the calculation of *W*_||_ the point-source Green’s function was approximated by a Gaussian distribution of length ${\bar{\sigma }}_{z}=0.3$ mm, and *W*_||_ includes the resistive wall and geometric changes in vacuum chamber components of NSLS-II.

**Figure 5:**
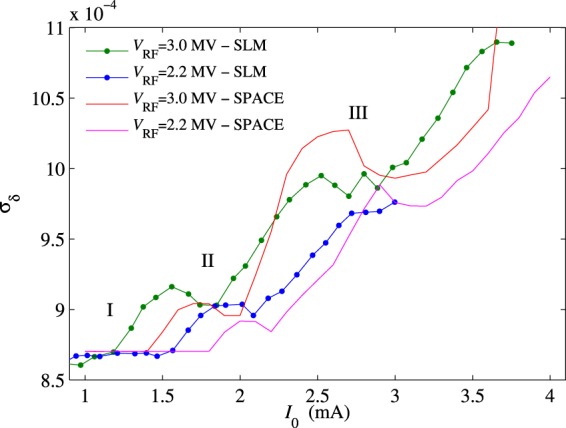
Comparison of the measured current-dependent energy spread to that simulated by the SPACE code at *V*_RF_ = 3.0 MV and *V*_RF_ = 2.2 MV.

**Figure 6:**
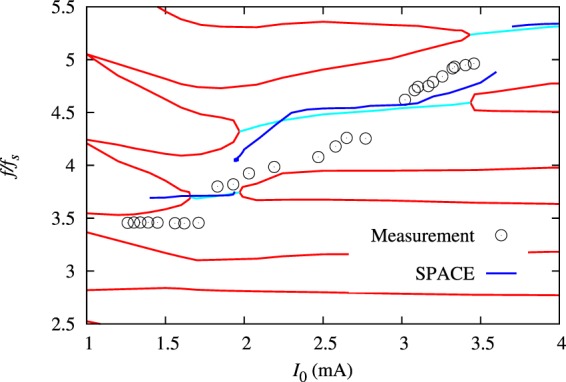
Current-dependent frequencies of longitudinal modes at *V*_RF_ = 3.0 MV. The black circles are the measured data, the dark blue lines are the frequency values calculated numerically using space, the cyan lines are the theoretically predicted frequencies of the unstable modes, which arise when two initially stable modes (red lines) merge.

Numerical simulations of the Vlasov-Fokker-Planck equation using space code are compared with SLM measurements. Figure 5 shows the ${\sigma }_{\delta }({I}_{0})$ dependence at ${V}_{{\rm{RF}}}=3$ MV (dark cyan - SLM, orange - space) and at ${V}_{{\rm{RF}}}=2.2$ MV (blue - SLM, magenta - space). At ${V}_{{\rm{RF}}}=3$ MV, for example, the thresholds labelled in Fig. 4 can be clearly seen in Fig. 5 as local minima in both the measured and simulated energy spread. A similar behavior is also observed at ${V}_{{\rm{RF}}}\mathrm{=2.2}$ MV, although the thresholds are less pronounced. From the analysis of the numerical results, we observed in general that local minima of *σ*_*δ*_ correspond to the currents at which the electron beam becomes less perturbed, with smaller fluctuations of energy spread about its new and inflated near-equilibrium *σ*_*δ*_. As shown in Figs. 4, 5 and 6, our numerical simulations provide a detailed picture of the measurements, and show that the impedance model gives a realistic description of the collective longitudinal physics at play.

To analyze the evolution of the energy spread above the first threshold and to investigate its modes of oscillation, we discuss now a theoretical framework based on the linearized Vlasov equation^28^, which interprets the longitudinal mode coupling as the physical mechanism responsible for the onset of the microwave instability.

The linearized Vlasov equation is obtained by neglecting the right hand side of Eq. (2), which is justified because the growth time of the microwave instability is much faster than the radiation damping time. Our linearization procedure is based on the expansion of the phase space density Ψ about a given two-dimensional Gaussian equilibrium density Ψ_0_, with perturbation Ψ_1_ assumed to have an harmonic time dependence with complex frequency Ω:

3 $${\rm{\Psi }}(q,\,p,\,\tau ;\,{I}_{0})={{\rm{\Psi }}}_{0}(q,\,p;\,{I}_{0})+{{\rm{\Psi }}}_{1}(q,\,p;\,{I}_{0}){e}^{-i\frac{{\rm{\Omega }}}{{\omega }_{s}}\tau }\mathrm{.}$$

The approximation of the equilibrium density with an intensity-dependent two-dimensional Gaussian distribution allows us to approximately take into account the effect of bunch lengthening and incoherent synchrotron frequency shift without the construction of the action-angle variables associated with the self-consistent equilibrium as was done in^29^. In addition, by assuming that above the *I*_*th*_ the energy spread increases to quench the instability, we can extend our analysis to the high intensity, microwave instability regime. The quenching mechanism can be understood as due to the additional Landau damping provided by the increase in energy spread.

In what follows we omit the explicit dependence of the bunch length *σ*_*z*_, synchrotron frequency *ω*_*s*_ and energy spread *σ*_*δ*_ on the bunch intensity *I*_0_. In this case, by linearizing about the Gaussian equilibrium density Ψ_0_ one can derive the following integral equation for the density bunching ${\mathcal B} (k)$ defined as the Fourier Transform of the longitudinal perturbation density ${\lambda }_{1}(q)=\int dp{{\rm{\Psi }}}_{1}(q,p)$^ 28^:

4 $$ {\mathcal B} (k)=\frac{2{I}_{0}}{\gamma {I}_{A}{\alpha }_{c}{\sigma }_{\delta }^{2}}\int d\kappa \frac{{Z}_{||}(\kappa )}{i\kappa {Z}_{0}} {\mathcal M} ({\sigma }_{z}k,\,{\sigma }_{z}\kappa ;\,{\rm{\Omega }}) {\mathcal B} (\kappa ),$$

where ${I}_{A}\approx 17\,kA$ is the Alfvén current, ${Z}_{0}\approx 377\,{\rm{\Omega }}$ is the free space impedance, $\gamma ={E}_{0}/{m}_{e}{c}^{2}$ is the Lorentz gamma factor, *m*_*e*_ is the electron rest mass and *c* is the velocity of light. The symmetric kernel ${\mathcal M}$ is given in terms of the modified Bessel function *I*_*n*_(*x*) by

5 $$\begin{array}{rcl} {\mathcal M} (x,y;\,{\rm{\Omega }}) & = & {e}^{-({x}^{2}+{y}^{2}\mathrm{)/2}}[{e}^{xy}-{I}_{0}(xy)]\\  &  & +{e}^{-({x}^{2}+{y}^{2}\mathrm{)/2}}\sum _{n\ge 1}\frac{\mathrm{2(}{\rm{\Omega }}/{\omega }_{s}{)}^{2}{I}_{n}(xy)}{{n}^{2}-{({\rm{\Omega }}/{\omega }_{s})}^{2}}\mathrm{.}\end{array}$$

In Eq. (4) *Z*_||_ is the Fourier Transform of the longitudinal wakefield *W*_||_.

The analysis leading to Eqs. (4, 5) is similar in some respects to that of^30,31^, but their work effectively neglected the RF focusing so that the synchrotron frequency *ω*_*s*_ played no explicit role, which is in sharp contrast to Eq. (5). On the other hand, both approaches result in nonlinear integral equations, which^30,31^ used to justify the so-called Boussard criterion^32^. The Boussard criterion gives an estimate for the bunched-beam *I*_*th*_ by applying the coasting beam theory of^33,34^ with the average bunch current being replaced by the peak current. While easy to compute, this criterion typically underestimates *I*_*th*_ by a factor of two or more.

Our approach to solve the problem involves considering solutions for a real frequency $\bar{{\rm{\Omega }}}$, in which case Eq. (4) can be understood as an eigenproblem for ${\mathcal B} (k)$ with eigenvalue $\propto {\sigma }_{\delta }^{2}/{I}_{0}$. Hence, at low current when the beam is stable, one can discretize Eq. (4) in the usual manner and apply standard eigenvalue solvers to determine at what current *I*_0_ a perturbation oscillates with scaled frequency $\bar{{\rm{\Omega }}}/{\omega }_{s}$. Doing this for a range of $\bar{{\rm{\Omega }}}$ shows that at a certain threshold current *I*_*th*_ two initially distinct frequencies merge, which we interpret as classical mode coupling as first described by Sacherer^13,14^. Hence, *I*_*th*_ gives the first microwave instability threshold, and unstable modes with complex frequencies Ω exist for *I*_0_ > *I*_*th*_.

This procedure to determine *I*_*th*_ is quite a bit different from the usual matrix solutions of, e.g.^35–37^, in which the perturbation is decomposed into orthogonal linear modes. One distinct advantage of our solution is that the matrix elements in the discretized version of Eq. (4) are given directly by the impedance, whereas in the usual matrix methods each matrix element is itself an integration of *Z*_||_ with some elementary function.

Our theory also differs from the nonlinear eigenvalue problem that was formulated and solved by in^38,39^, where their approach resolves certain convergence issues of previous methods while incorporating the change in the bunch profile with *I*_0_ in a fully self-consistent manner. We deal with the latter using a very simple approximation that sets the synchrotron frequency according to the rms bunch length *σ*_*z*_, so that *ω*_*s*_ in Eq. (4) is to be given by ${\omega }_{s}=c{\alpha }_{c}{\sigma }_{\delta }/{\sigma }_{z}$. While the theory employed here is only approximate, we find that it is relatively easy to apply and appears to give a useful physical interpretation of the microwave instability threshold currents in terms of a succession of mode coupling events.

To understand this final statement, we extend the application of the theoretical equations (4),(5) to currents above the threshold by increasing the energy spread *σ*_*δ*_ so as to just quench the instability. From the prefactor of Eq. (4) this implies that ${\sigma }_{\delta }({I}_{0})={\sigma }_{\delta }\mathrm{(0)}\sqrt{{I}_{0}/{I}_{th}}$, allowing us to ignore the variation of *σ*_*z*_ with current. On the other hand, if we include the bunch lengthening with *I*_0_ we find that there exists another threshold current at which the mode coupling occurs with frequency close to $\bar{{\rm{\Omega }}}+{\omega }_{s}$. In other words, as the current increases above the first microwave instability threshold, the increase in bunch length results in a higher-order mode coupling which then becomes the most unstable mode, and the theory identifies these higher-order mode coupling events with the higher-order thresholds.

To confirm these conclusions, Fig. 6 presents the normalized frequencies $f/{f}_{s}$ of longitudinal modes vs. *I*_0_ at *V*_RF_ = 3 MV combining the measured, numerically simulated, and semi-analytically calculated data. The stable modes of oscillation calculated analytically with Eqs. (4,5) are represented by the red lines, while the unstable modes are represented by the cyan lines (analytical), the blue lines (space simulations), and the circles (measurements). As it can be seen in Fig. 6, above the first microwave instability threshold, all three data sets show the appearance of an unstable mode with the frequency between $3{f}_{s}$ and $4{f}_{s}$ at *V*_RF_ = 3 MV. Based on the applied theory, the appearance of an unstable mode at $3.7{f}_{s}$ (observed numerically and analytically) results from the coupling of two modes that can oscillate incoherently below the first microwave instability threshold. However we found the detection of those stable modes, both from particle tracking simulations and beam measurements, very challenging without a special excitation mechanism, which needs to be further investigated.

We studied dynamic properties of the energy spread above the microwave instability threshold, in regimes characterized by states of quasi-equilibrium and by the presence of one or several stable modes of oscillation. The beam dynamics has been studied experimentally by high-precision measurements performed at the NSLS-II storage ring with different machine settings. The measurements carried out with three distinct diagnostic methods, show very clear and reproducible intensity-dependent patterns, thus allowing a theoretical interpretation. To this end, we performed numerical simulations of the Vlasov-Fokker-Plank equation and compared the results with a novel eigenvalue analysis based on the linearized Vlasov equation. This analysis, with the assumption that two stable oscillation modes merge together at the instability threshold, allows us to interpret the microwave instability as a classical mode coupling. Moreover, by assuming that the energy spread above threshold increases to provide sufficient Landau damping to suppress the growth of unstable modes, the full spectral analysis is reduced to the determination of solely stable modes of oscillation. The measurements discussed in this paper, together with the methods used for their interpretation, offer an opportunity to predict and further characterize the microwave instability threshold, and represent an effort to enhance the understanding of the dynamic evolution of the energy spread above the threshold which may further enable controlled operation of electron storage rings at high current.
